# Glucocorticoids as cytokine inhibitors: role in neuroendocrine control and therapy of inflammatory diseases

**DOI:** 10.1155/S0962935193000365

**Published:** 1993

**Authors:** Giamila Fantuzzi, Pietro Ghezzi

**Affiliations:** “Mario Negri” Institute for Pharmacological Research via Eritrea 62 Milano 20157 Italy

## Abstract

Glucocorticoids are potent inhibitors of inflammation and endotoxic shock. This probably occurs through an inhibition of the synthesis of pro-inflammatory cytokines as well as of many of their toxic activities. Therefore, endogenous glucocorticoids (GC) might represent a major mechanism in the control of cytokine mediated pathologies. GC inhibit the synthesis of cytokines in various experimental models. Adrenalectomy or GC antagonists potentiate TNF, IL-1 and IL-6 production in LPS treated mice. GC inhibit the formation of arachidonic acid metabolites and the induction of NO synthase. They also inhibit various activities of cytokines including toxicity, haemodynamic shock and fever. Adrenalectomy sensitizes to the toxic effects of LPS, TNF and IL-1. On the other hand, GC potentiate the synthesis of several cytokine induced APP by the liver. Since many of these proteins have anti-toxic activities (antioxidant, antiprotease etc.) or bind cytokines, this might well represent a GC mediated protective feedback mechanism involving the liver. Not only do GC inhibit cytokines, but *in vivo* LPS and various cytokines (TNF, IL-1, IL-6) increase blood GC levels through a central mechanism involving the activation of the HPA. Thus, this neuroendocrine response to cytokines constitutes an important immunoregulatory feedback involving the brain.


Mediators of Inflammation 2, 263–270 (1993)

### Glucocorticoids as cytokine inhibitors: role in neuroendocrine control and therapy of inflammatory diseases

Giamila Fantuzzi and Pietro Ghezzi[CA]

"Mario Negri" Institute for Pharmacological Research, via Eritrea 62, 20157 Milano, Italy

[CA] Corresponding Author

**GLUCOCORTICOIDS are potent inhibitors of inflammation and endotoxic shock. This probably occurs through an inhibition of the synthesis of pro-inflammatory cytokines as well as of many of their toxic activities. Therefore, endogenous glucocorticoids (GC) might represent a major mechanism in the control of cytokine mediated pathologies. GC inhibit the synthesis of cytokines in various experimental models. Adrenalectomy or GC antagonists potentiate TNF, IL-1 and IL-6 production in LPS treated mice. GC inhibit the formation of arachidonic acid metabolites and the induction of NO synthase. They also inhibit various activities of cytokines including toxicity, haemodynamic shock and fever. Adrenalectomy sensitizes to the toxic effects of LPS, TNF and IL-1. On the other hand, GC potentiate the synthesis of several cytokine induced APP by the liver. Since many of these proteins have anti-toxic activities (antioxidant, antiprotease etc.) or bind cytokines, this might well represent a GC mediated protective feedback mechanism involving the liver. Not only do GC inhibit cytokines, but *in vivo* LPS and various cytokines (TNF, IL-1, IL-6) increase blood GC levels through a central mechanism involving the activation of the HPA. Thus, this neuroendocrine response to cytokines constitutes an important immunoregulatory feedback involving the brain.**

**Key words:** Cytokines, Endotoxic shock, Glucocorticoids, Inflammation, Neuroendocrine control

### Introduction

Glucocorticoids (GC) are effective in the treatment of a variety of inflammatory and autoimmune diseases and in these fields much of current attention is focused on cytokines. This review discusses the effects of GC on the production and actions of cytokines in order to obtain a better understanding of how the anticytokine action of GC is involved in their pharmacological activity. This review focuses on the cytokines primarily involved in the regulation of inflammatory processes, IL-1, IL-6, IL-8 and TNF.

Most studies of pharmacological modulation of cytokines have used animal models of endotoxaemia. In fact, endotoxin (lipopolysaccharide, LPS) is a potent and widely used inducer of cytokine production *in vivo* and on cultured cells, and its toxic effects are antagonized by inhibitors or antagonists of IL-1 or TNF.[1–3]

The role of GC in the control of cytokine mediated effects of LPS has been demonstrated by two different approaches: removal of endogenous GC (by adrenalectomy or GC-receptor antagonists), or administration of exogenous GC. Adrenalectomy increases sensitivity to the lethal action of LPS[4,5] and identical results have been obtained with the GC receptor antagonist mifepristone (RU38486).[6,7] Treatment with GC markedly protects against LPS toxicity.[8–10] Susceptibility to the lethal action of LPS[11] and TNF[12] varies with the circadian rhythm; however, the role of endogenous GC is far from being established. Biochemically, GC inhibit the LPS induced production of inflammatory mediators, such as arachidonic acid metabolites and nitric oxide (NO). It is therefore important to define which of these actions are due to inhibition of cytokine production and which might involve a direct inhibition of the cytokines' action.

### Inhibition of cytokine production

*Exogenous glucocorticoids:* Most of the *in vitro* studies focused on IL-1 and TNF, whose induction is suppressed by various GC (Table 1). IFN$\gamma$ can partly overcome GC suppression of TNF production *in vitro* by mouse peritoneal macrophages.[26] In contrast, IL-4 enhances the inhibitory action of GC on IL-1, TNF and prostaglandins (PG) $E_2$ production by human monocytes.[27] However, GC had no inhibitory effects when phorbol myristate acetate (PMA) was used as a stimulus for TNF, IL-1 or IL-8 production.[17,21,28]

The inhibitory effect of GC on IL-1 production may proceed through the inhibition of IL-1 gene transcription and a decrease in the stability of

© 1993 Rapid Communications of Oxford Ltd

**Table 1.** Inhibition of proinflammatory cytokine production by glucocorticoids *in vitro*

| Cytokine | Inducer | References |
|---|---|---|
| IL-1 | LPS, *S. aureus*, IL-1 | 13–15 |
| TNF | LPS, anti-IgG | 16, 17 |
| IL-6 | LPS, IL-1, TNF | 18, 19 |
| IL-8 | LPS, IL-1, TNF, cAMP | 19–21 |
| IL-2 | IL-1 | 22 |
| GM-CSF | IL-1, TNF | 19, 23 |
| G-CSF | IL-1, TNF | 23, 24 |
| MCP-1 | IL-1 | 25 |

IL-interleukin; TNF, tumour necrosis factor; GM-CSF, granulocyte-macrophage colony-stimulating factor; G-CSF, granulocyte colony-stimulating factor; MCP-1, monocyte chemotactic protein-1.

IL-1 mRNA.[29] The fact that GC did not inhibit PMA induced IL-1 might be explained by postulating that PMA has a stabilizing effect on IL-1 mRNA.[28] The inhibitory effect of GC on IL-1$\beta$ gene expression was reversed by inhibitors of protein synthesis.[29] This has some analogy with other anti-inflammatory activities of GC that require the synthesis of lipocortins.[30]

Cytokines often induce each other, in a cascade fashion, and GC inhibit IL-1 and TNF induction of various cytokines (Fig. 1). In mice or rats treated with LPS or anti-CD3 antibodies GC inhibited the production of circulating TNF, IL-1, IL-6 and IFN$\gamma$.[31–34] TNF seems to be more sensitive to GC inhibition *in vivo* than IL-1 or IL-6.[35,36]

GC inhibit TNF and IL-6 induced by stimuli other than LPS. Dexamethasone (DEX) inhibited TNF induction in mice after partial hepatectomy,[37] and IL-1 in a rat model of zymosan induced pleurisy.[38] It also lowered serum IL-6 levels in adjuvant arthritis in rats.[39]

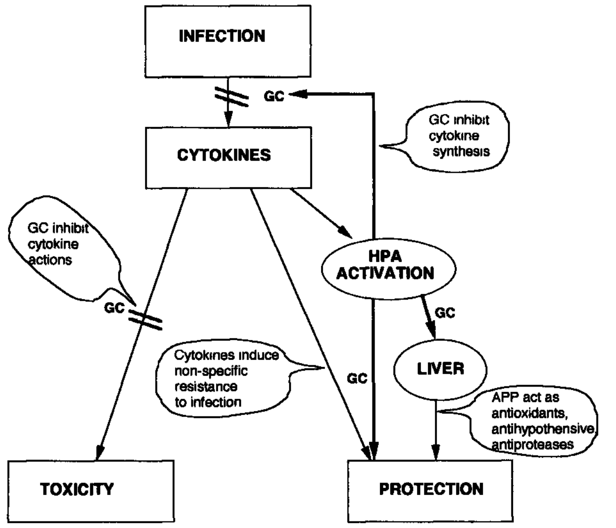

FIG. 1. Dual role of cytokines in infection and inflammation. Stimulation of neuroendocrine system, by increasing endogenous GC, inhibits cytokine production and potentiates cytokine induced protective responses. The protective mechanism involving stimulation of APP synthesis by the liver is depicted.

In human volunteers giving an injection of LPS, GC inhibited IL-6 and TNF production.[40,41] DEX, administered to IL-2 treated cancer patients[42] or to patients undergoing cardiopulmonary bypass,[43] reduced the TNF production observed in these conditions.

*Ex vivo* studies on cells from patients with sarcoidosis,[44] Crohn's disease[45] or rheumatoid arthritis[46] found decreased IL-1, IL-6 and IL-8 production after GC treatment.

*Effects of adrenalectomy or glucocorticoid antagonists:* The studies mentioned above employed exogenous GC. More direct evidence of the inhibitory effect of endogenous GC comes from studies where animals were adrenalectomized (ADX) or treated with GC antagonists. Adrenalectomy sensitizes to the lethal action of infections, LPS,[4,5] turpentine[47] or complete Freund's adjuvant.[48] This was related to increased production of TNF and IL-1.[49]

GC might be important in switching off TNF production and, in fact, in ADX mice injected with LPS, TNF levels remained elevated up to 5 h, whereas in sham-operated mice they returned to zero by 3 h.[50] This prolonged induction was not associated with higher peak TNF levels. In another study, however,[51] ADX mice had higher peak (1.5 h) TNF levels, but the kinetics of TNF induction was similar to that of intact mice, returning to zero within 4 h. Unpublished data from our laboratory agree with the latter report.

Production of IL-1 was also increased *ex vivo*, in peritoneal macrophages from ADX rats.[52] The authors' group reported that intracerebroventricular (i.c.v.) injection of IL-1 in rats induced high circulating levels of IL-6, and this effect was potentiated by adrenalectomy.[53] The data on ADX animals were confirmed by using RU38486 which is reported to increase LPS toxicity and induction of TNF and IL-6.[6,7] RU38486 also increased mortality in a mouse model of septic peritonitis.[54]

### Effects on cytokine receptors

Glucocorticoids increase the expression of IL-1 receptors in B-lymphocytes, fibroblasts, and some cell lines[55,56] but not in T-lymphocytes, large granular lymphocytes or monocytes. While this increase in IL-1 receptors might seem paradoxical, in view of the anti-inflammatory action of GC, it could result in a change in IL-1 distribution *in vivo*, where IL-1 might be soaked up by some cells rather than others.

This was also suggested in the case of IL-6 whose receptor is markedly decreased by GC in monocytes but not in hepatocytes. However, GC appeared to be essential for the induction of hepatocyte IL-6 receptors by IL-1 and IL-6 itself, and it was suggested that monocytes are normally involved in binding trace amounts of IL-6, thus preventing its interaction with the hepatocyte.[57]

Information on TNF receptors is scant; DEX reduced the number of 75 kDa TNF receptors on U937 cells[58] but did not affect the number of TNF binding sites on mouse hepatocytes in DEX treated mice.[59]

### Effects on cytokine actions

Cytokines have a variety of actions on different cells, resulting in various pathological effects associated with infective or inflammatory diseases.[60] The effects of anti-inflammatory agents, including GC and non-steroidal anti-inflammatory drugs, on the activities of TNF and IL-1 have been amply documented. Here the effect of GC on various activities of cytokines, *in vivo* and *in vitro* is reviewed.

In general, GC inhibit the pro-inflammatory and toxic effects of cytokines. These effects belong mainly to IL-1 and TNF, whose role as mediators of the mortality associated with Gram-negative septicaemia has been demonstrated by the protective effect of specific IL-1 and TNF inhibitors.[2,3,6] On the other hand, GC have been reported to potentiate the induction of acute phase proteins (APP).

*Effects on inflammation:*

*Arachidonate metabolism.* Prostaglandins and other arachidonic acid oxygenation products are major mediators of inflammation. Their production is stimulated by IL-1,[61] and this was suggested to be an important mechanism in the inflammatory and pyrogenic action of IL-1.

Through GC induced lipocortin, GC inhibit the activity and synthesis of $PLA_2$,[62] the enzyme that provides free arachidonic acid for PG synthesis. IL-1, TNF and IFN$\gamma$ induce PG synthesis by stimulating $PLA_2$ activity, and GC could inhibit this.[63–65] This suggests that the inhibitory effect of GC is at the level of $PLA_2$, since arachidonic acid induced PG production was not reduced.[63] Furthermore, GC inhibit the *de novo* synthesis of cyclooxygenase induced by IL-1, TNF or LPS.[66–68]

$PLA_2$ is not implicated only in the inflammatory action of cytokines but also in the tumoricidal activity of TNF. GC inhibit the cytotoxic activity of TNF on tumour cells *in vitro*[69,70] as well as its tumoricidal activity *in vivo*[71] and it was suggested this was due to inhibition of $PLA_2$. Interestingly, this activity of GC seems not to be mediated by lipocortin.[72]

*Chemotaxis and adherence.* Pretreatment with DEX abolished the neutrophil migration induced by injection of IL-8 or IL-1 in rats or mice[73–75] thus suggesting that GC may directly inhibit IL-8 chemotactic activity, in addition to suppressing IL-1 induced IL-8 production. The inhibitory effect of GC on IL-1 induced chemotaxis seems to be mediated by the induction of lipocortin which, in fact, inhibits IL-1 induced chemotaxis, and anti-lipocortin antibodies abolished the inhibitory action of GC.[62] Contrasting indications were obtained on the effect of GC on cytokine induced expression of the adhesion molecule ICAM-1.[76,77]

*Nitric oxide pathway.* Nitric oxide has been identified as a major mediator of shock and inflammation. Its production is increased by LPS, TNF, IL-1 and IFN$\gamma$ through the induction of NO synthase.[78] GC inhibited this induction by cytokines and LPS[79,80] while not affecting its enzymatic activity.[81]

*Toxic, behavioural and catabolic effects:* The most impressive evidence for a protective role of endogenous GC against the toxic effects of cytokines comes from studies reporting that ADX mice are sensitized to the lethal effects of IL-1 and TNF;[52,82] thus their increased sensitivity to LPS is not only due to increased cytokine production (as discussed above) but also to increased sensitivity to their actions. Similar results have been obtained with RU38486.[83] Furthermore, DEX protected against the lethal effect of a high dose of TNF in intact mice without affecting its clearance.[59,71]

The well-known haemodynamic effects of GC might also be important in protecting against the toxicity of IL-1 and TNF, which cause a marked and often lethal hypotension.[84]

GC might regulate other physiological responses to infection too. For instance, ADX rats are more susceptible to LPS induced fever[85] and GC attenuated the sleep response to bacterial infection in rabbits.[86] However, it is not clear whether the effects of GC in these models are due to inhibition of the production of cytokines or of their action.

Another well-known central effect of IL-1 and TNF is anorexia. Contrasting results were reported; one paper reported that DEX pre-treatment blocked the suppression of food intake induced by i.c.v. IL-1 administration in rats,[87] while another showed that IL-1 induced anorexia was less pronounced in ADX rats.[88]

*Effects on acute phase proteins:* Induction of hepatic APP is part of a reorientation of the liver metabolism which includes an increase in the synthesis of APP and a decrease in negative acute phase reactants. GC are important for the induction of several APP, and are required for the production of fibrinogen by cultured hepatocytes stimulated with crude leukocytic extract.[89] The permissive action of GC was also reported *in vivo* where inflammatory stimuli caused little or no induction of angiotensinogen,[90] haptoglobin[47] and $\alpha_2$-macrofetoprotein[91] in ADX

animals. Among the various cytokines, those that play a key role as hepatocyte stimulating factors include IL-6, leukaemia inhibitory factor (LIF) and oncostatin M (OM). Induction of most APP in hepatoma cells stimulated *in vitro* with recombinant preparations of these cytokines was potentiated by GC.[92–94]

Induction of APP could be part of the protective feedback response to infection and inflammation. Some APP have activities that might be 'protective', either by binding cytokines, like $\alpha_2$-macroglobulin;[95] by maintaining blood pressure, like angiotensinogen;[90] or acting as antioxidants or proteinase inhibitors. The protective role of APP is elegantly demonstrated by Xia and Samols[96] who showed that transgenic mice overexpressing rabbit C-reactive protein were protected against LPS or IL-1/TNF induced lethality.

As mentioned above, some 'normal liver proteins' behave as negative acute phase reactants, including albumin and cytochrome P-450. While this can be explained on the basis of cell economy, depression of cytochrome P-450 may have a functional role. In fact, cytochromes of the P-450 family are also implicated in steroidogenesis and TNF inhibited ACTH induced steroidogenic cytochrome P-450, resulting in decreased GC production.[97] This effect might limit the increase in blood GC by TNF described below. It must also be noted that cytochromes of the P-450 family are implicated in the metabolism of arachidonic acid, thus suggesting another possible involvement in the regulation of inflammation.

### Effect of cytokines on glucocorticoid receptors and action

Glucocorticoids are not only cytokine inhibitors but, the other way round, cytokines may counteract some effects of GC. As early as 1976, Moore *et al.*[98,99] showed that GC induction of some enzymes was inhibited by LPS; they identified a macrophage product that was termed GAF (glucocorticoid antagonizing factor). LPS also downregulates hepatic GC receptors,[100] an effect that is tissue specific since LPS had the opposite effect in macrophages.[101] Although the molecular structure of GAF has never been identified it is conceivably one of the several activities of IL-1, IL-6, TNF or other known cytokines. In this respect, it is interesting to note that IL-1 decreases hepatic GC receptors thus inhibiting the induction of phosphoenolpyruvate carboxykinase.[102]

### Cytokine activation of the hypothalamus–pituitary–adrenal axis

Inflammation and injection of LPS induce a rise in blood GC, due to activation of the hypothalamus–pituitary–adrenal axis (HPA).[103] LPS induced GC secretion *in vivo* is inhibited by selective depletion of macrophages, suggesting that macrophage derived cytokines mediate the effect of LPS.[104] In fact, treatment of mice with IL-1 increased blood ACTH and GC levels[105] and anti-IL-1 receptor antibodies blocked LPS induced ACTH release.[106] While an earlier work reported that TNF had no such effect, TNF and IL-6 also increased blood GC, although they were less potent than IL-1.[107,108]

Although in different experimental models *in vitro*, IL-1 and IL-6 directly stimulated the release of ACTH from pituitary cells,[109,110] the increase of blood GC observed *in vivo* seems mainly due to a stimulation of hypothalamic cells to release CRF.[111] IL-1 induced GC was associated with increased CRF levels in the pituitary portal vessels and anti-CRF antibodies inhibited IL-1 induced GC, thus indicating that IL-1 exerts this effect on the hypothalamus.[112,113]

A series of papers focused on the role of PG in IL-1 activation of the HPA. The cyclooxygenase inhibitor, indomethacin, inhibited CRF release *in vitro*[114] as well as activation of the HPA *in vivo*.[115,116]

Stress induced activation of the HPA is downregulated by a feedback mechanism, and inhibited by DEX pre-treatment. In comparison, IL-1 induced HPA activation is less sensitive to the inhibitory effect of DEX. As a possible explanation for this difference, it was reported that IL-1 decreases hippocampal GC receptors thus desensitizing them to DEX effects.[117]

*HPA in the regulation of immunity, autoimmunity and inflammation:*

*Immunity.* Since GC are potent inhibitors of cytokine production, cytokine activation of HPA might obviously constitute a negative feedback mechanism that regulates their production.[105] To support this theory, it was reported that hypophysectomized animals produce more IL-1, IL-6 and TNF.[49,53] Activation of HPA by i.c.v. injected IL-1 resulted in suppression of spleen macrophage IL-1 production *ex vivo*, which was not observed in ADX mice.[118]

Another effect of central administration of IL-1 is to lower the peripheral cellular immune response in terms of natural killer cell activity, lymphocyte response to mitogens and IL-2 production.[119,120] These effects were blunted by anti-CRF antibodies and were less pronounced in ADX animals, indicating that HPA is to some extent involved.

*Autoimmune diseases.* Experimental allergic encephalomyelitis (EAE) is a demyelinating disease induced by immunization to myelin basic protein, and is used as an animal model for multiple sclerosis. Recent data have indicated a pathogenetic role of TNF in this disease, as demonstrated by the protective effect of anti-TNF antibodies.[121] The role of endogenous GC in the susceptibility to the disease has been reviewed by Mason.[122] EAE activates the HPA as revealed by the increase in serum GC. ADX rats do not recover spontaneously from EAE, and adrenalectomy increases the mortality rate in this animal model. Rat strains that are genetically resistant to the development of EAE have more marked HPA activation to stressing stimuli, and adrenalectomy can render them normally susceptible to EAE. These data on the HPA regulation of EAE provide a rationale for the use of GC in the therapy of multiple sclerosis.

*Inflammation.* Since GC are potent anti-inflammatory agents, HPA activation may also function to limit inflammatory processes. TNF and IL-1 were reported to have anti-inflammatory action in carrageenan or dextran induced hind paw oedema in rats.[123] This was due to the IL-1 and TNF induced rise in GC, and was not observed in ADX rats.

Lewis rats, which are genetically prone to streptococcal cell wall (SCW) induced arthritis, had markedly impaired ACTH and GC responses to SCW or IL-1.[124] On the other hand, RU38486 induced susceptibility to SCW arthritis in normal rats. Thus genetic susceptibility to arthritis might be related to a defective HPA responsiveness to cytokines.

### Glucocorticoid therapy in cytokine mediated pathologies

In view of their potent anti-cytokine action, GC might theoretically constitute drugs of choice in the therapy of cytokine mediated pathologies, i.e. those where a pathogenetic role of cytokines has been demonstrated in animal models by the protective action of anti-cytokine antibodies. The earliest works of passive immunization of mice against TNF showed that this cytokine is the key mediator of mortality induced by LPS or Gram-negative sepsis.[2,3] As mentioned above, GC has a strong protective effect in mice treated with lethal doses of LPS[8–10] and septic shock was an indication for the use of high GC doses.[125]

However, in clinical trials GC did not reduce mortality in septic shock patients, and their indication for use in this condition was withdrawn by the FDA.[126,127] This discrepancy between animal and clinical studies might have various reasons. For instance, in most studies GC were administered to animals before or concomitantly with LPS, a situation that is quite different from a septic patient. Furthermore, in septic shock patients, Gram-negative sepsis is often associated with Gram-positive infection,[128] and in mice where polymicrobial sepsis was induced by caecal ligation and puncture, anti-TNF antibodies and GC tended to increase mortality.[129,130] These data indicate the existence of a complex pathway where one must consider the balance between the pathogenetic, toxic activities of cytokines and their protective actions against infection.[131]

A different picture emerges on the role of TNF and IL-1 in the pathogenesis of meningitis,[132,133] where animal studies have shown a protective effect of GC. In meningitis patients, antibiotic therapy would result in bacterial cell lysis and consequent liberation of LPS which would trigger the cytokine cascade.[134] It is therefore a current therapeutic approach, supported by clinical trials, to administer DEX 30 min before antibiotics to inhibit cytokine production, thus preventing an important component of the pathology.

Thus, glucocorticoids inhibit cytokines at multiple sites. However, in most animal models they are effective when given as pre-treatments, they aspecifically inhibit the synthesis of all cytokines, and they are also potent immunosuppressive agents. This limits their effectiveness in the therapy of cytokine mediated pathologies where a balance of toxic versus protective effects of cytokines is likely to exist.

### References

1. Dinarello CA. Interleukin-1 and interleukin-1 antagonism. *Blood* 1991; **77**: 1627–1652.
2. Beutler B, Milsark IW, Cerami AC. Passive immunization against cachectin/tumour necrosis factor protects mice from lethal effect of endotoxin. *Science* 1985; **229**: 869–871.
3. Tracey KJ, Fong Y, Hesse DG, *et al.* Anti-cachectin/TNF monoclonal antibodies prevent septic shock during lethal bacteraemia. *Nature* 1987; **330**: 662–664.
4. Abernathy RS, Halberg F, Spink WW. Studies on the mechanism of chlorpromazine protection against *Brucella* endotoxin in mice. *J Lab Clin Med* 1957; **49**: 708–715.
5. Swingle WW, Remington JW. Role of adrenal cortex in physiological processes. *Physiol Rev* 1944; **24**: 89–127.
6. Lazar G, Agarwal MK. The influence of a novel glucocorticoid antagonist on endotoxin lethality in mice strains. *Biochem Med Metab Biol* 1986; **36**: 70–74.
7. Hawes AS, Rock CS, Keogh CV, Lowry SF, Calvano SE. *In vivo* effects of the antiglucocorticoid RU 486 on glucocorticoid and cytokine responses to *Escherichia coli* endotoxin. *Infect Immun* 1992; **60**: 2641–2647.
8. Berry LJ, Smythe DS. Effects of bacterial endotoxins on metabolism. VII. Enzyme induction and cortisone protection. *J Exp Med* 1964; **120**: 721–732.
9. Gadina M, Bertini R, Mengozzi M, Zandalasini M, Mantovani A, Ghezzi P. Protective effect of chlorpromazine on endotoxin toxicity and TNF production in glucocorticoid-sensitive and glucocorticoid-resistant models of endotoxic shock. *J Exp Med* 1991; **173**: 1305–1310.
10. Evans GE, Zuckerman SH. Glucocorticoid-dependent and -independent mechanisms involved in lipopolysaccharide tolerance. *Eur J Immunol* 1991; **21**: 1973–1979.
11. Halberg F, Johnson EA, Brown BW, Bittner JJ. Susceptibility rhythm to *E. coli* endotoxin and bioassay. *Proc Soc Exp Biol* 1960; **103**: 142–147.
12. Langevin T, Young J, Walker K, Roemeling R, Nygaard S, Hrunhesky WJM. The toxicity of tumour necrosis factor (TNF) is reproducibly different at specific times of the day. *Proc Am Assoc Cancer Res* 1987; **28**: 398.
13. Arend WP, Massoni RJ. Characteristics of bacterial lipopolysaccharide induction of interleukin-1 synthesis and secretion by human monocytes. *Clin Exp Immunol* 1986; **64**: 656–664.
14. Knudsen PJ, Dinarello CA, Strom TB. Glucocorticoids inhibit transcriptional and post-transcriptional expression of interleukin-1 in U937 cells. *J Immunol* 1987; **139**: 4129–4134.
15. Ghezzi P, Dinarello CA. Interleukin-1 induces interleukin-1. III. Specific inhibition of IL-1 production by IFN-γ. *J Immunol* 1988; **140**: 4238–4244.

16. Beutler B, Krochin N, Milsark IW, Luedke C, Cerami A. Control of cachectin (tumour necrosis factor) synthesis: mechanisms of endotoxin resistance. *Science* 1986; **232**: 977–980.
17. Debets JMH, Ruers TJM, Van der Linden MPMH, Van der Linden CJ, Buurman WA. Inhibitory effect of corticosteroids on the secretion of tumour necrosis factor (TNF) by monocytes is dependent on the stimulus inducing TNF synthesis. *Clin Exp Immunol* 1989; **78**: 224–229.
18. Waage A, Slupphaug G, Shalaby R. Glucocorticoids inhibit the production of IL6 from monocytes, endothelial cells and fibroblasts. *Eur J Immunol* 1990; **20**: 2439–2443.
19. Tobler A, Meier R, Seitz M, Dewald B, Baggiolini M, Fey MF. Glucocorticoids downregulate gene expression of GM-CSF, NAP-1/IL-8 and IL-6, but not of M-CSF in human fibroblasts. *Blood* 1992; **79**: 45–51.
20. Brown Z, Strieter RM, Chensue SW, *et al.* Cytokine-activated human mesangial cells generate the neutrophil chemoattractant, interleukin 8. *Kidney Int* 1991; **40**: 86–90.
21. Anttila HSI, Reitamo S, Ceska M, Hurme M. Signal transduction pathways leading to the production of IL-8 by human monocytes are differentially regulated by dexamethasone. *Clin Exp Immunol* 1992; **89**: 509–512.
22. Tracey DE, Hardee MM, Richard KA, Paslay JW. Pharmacological inhibition of interleukin-1 activity on T-cells by hydrocortisone, cyclosporine, prostaglandins and cyclic nucleotides. *Immunopharmacology* 1988; **15**: 47–62.
23. Hamilton JA, Piccoli DS, Cebon J, *et al.* Cytokine regulation of colony-stimulating factor (CSF) production in cultured human synovial fibroblasts. II. Similarities and differences in the control of interleukin-1 induction of granulocyte-macrophage CSF and granulocyte-CSF production. *Blood* 1992; **79**: 1413–1419.
24. Kerner B, Teichmann B, Welte K. Dexamethasone inhibits tumour necrosis factor-induced granulocyte colony-stimulating factor production in human endothelial cells. *Exp Hematol* 1992; **20**: 334–338.
25. Villiger PM, Terkeltaub R, Lotz M. Monocyte chemoattractant protein-1 (MCP-1) expression in human articular cartilage. Induction by peptide regulatory factors and differential effects of dexamethasone and retinoic acid. *J Clin Invest* 1992; **90**: 488–496.
26. Luedke CE, Cerami A. Interferon-$\gamma$ overcomes glucocorticoid suppression of cachectin/tumour necrosis factor biosynthesis by murine macrophages. *J Clin Invest* 1990; **86**: 1234–1240.
27. Hart PH, Whitty GA, Burgess DR, Croatto M, Hamilton JA. Augmentation of glucocorticoid action on human monocytes by interleukin-4. *Lymphokine Res* 1990; **9**: 147–153.
28. Hurme M, Siljander P, Anttila H. Regulation of interleukin-1$\beta$ production by glucocorticoids in human monocytes: the mechanism of action depends on the activation signal. *Biochem Biophys Res Commun* 1991; **180**: 1383–1389.
29. Lee SW, Tsou A-P, Chan H, *et al.* Glucocorticoids selectively inhibit the transcription of the interleukin 1$\beta$ gene and decrease the stability of interleukin 1$\beta$ mRNA. *Proc Natl Acad Sci USA* 1988; **85**: 1204–1208.
30. Hiralta F, Schiffman D, Venkatasubramanian K, Salomon D, Axelrod J. A phospholipase $A_2$ inhibitory protein in rabbit neutrophils induced by glucocorticoids. *Proc Natl Acad Sci USA* 1980; **77**: 2533–2536.
31. Ulich TR, Guo K, Irwin B, Remick DG, Davatelis GN. Endotoxin-induced cytokine gene expression *in vivo*. II. Regulation of tumour necrosis factor and interleukin-1$\alpha/\beta$ expression and suppression. *Am J Pathol* 1990; **137**: 1173–1185.
32. Staruch MJ, Wood DD. Reduction of serum interleukin-1-like activity after treatment with dexamethasone. *J Leuk Biol* 1985; **37**: 193–207.
33. Waage A. Production and clearance of tumour necrosis factor in rats exposed to endotoxin and dexamethasone. *Clin Immunol Immunopathol* 1987; **45**: 348–355.
34. Ferran C, Dy M, Merite S, *et al.* Reduction of morbidity and cytokine release in anti-CD3 MoAb-treated mice by corticosteroids. *Transplantation* 1990; **50**: 642–648.
35. Zuckerman SH, Evans GF, Butler LD. Endotoxin tolerance: independent regulation of interleukin-1 and tumour necrosis factor expression. *Infect Immun* 1991; **59**: 2774–2780.
36. Sironi M, Gadina M, Kankova M, *et al.* Differential sensitivity of *in vivo* TNF and IL-6 production to modulation by antiinflammatory drugs in mice. *Int J Immunopharmacol* 1992; **14**: 1045–1050.
37. Satoh M, Adachi K, Suda T, Yamazaki M, Mizuno D. TNF-driven inflammation during mouse liver regeneration after partial hepatectomy and its role in growth regulation of liver. *Mol Biother* 1991; **3**: 136–147.
38. Perretti M, Solito E, Parente L. Evidence that endogenous interleukin-1 is involved in leukocyte migration in acute experimental inflammation in rats and mice. *Agents & Actions* 1992; **35**: 71–78.
39. Theisen-Popp P, Pape H, Muller-Peddinghaus R. Interleukin-6 (IL-6) in adjuvant arthritis of rats and its pharmacological modulation. *Int J Immunopharmacol* 1992; **14**: 565–571.
40. Rock CS, Coyle SM, Keogh CV, *et al.* Influence of hypercortisolemia on the acute-phase protein response to endotoxin in humans. *Surgery* 1992; **112**: 467–474.
41. Barber AE, Coyle SM, Marano MA, *et al.* Glucocorticoid therapy alters hormonal and cytokine responses to endotoxin in man. *J Immunol* 1993; **150**: 1999–2006.
42. Mier JW, Vachino G, Klempner MS, *et al.* Inhibition of interleukin-2-induced tumour necrosis factor release by dexamethasone: prevention of an acquired neutrophil chemotaxis defect and differential suppression of interleukin-2-associated side effects. *Blood* 1990; **76**: 1933–1940.
43. Jansen NJ, van Oeveren W, van der Broek L, *et al.* Inhibition by dexamethasone of the reperfusion phenomena in cardiopulmonary bypass. *J Thorac Cardiovasc Pulmonary Surg* 1991; **102**: 515–525.
44. Zabel P, Horst HJ, Kreiler C, Schlaak M. Circadian rhythm of interleukin-1 production of monocytes and the influence of endogenous and exogenous glucocorticoids in man. *Klin Wochenschr* 1990; **68**: 1217–1221.
45. Andus T, Gross V, Casar I, *et al.* Activation of monocytes during inflammatory bowel disease. *Pathobiology* 1991; **59**: 166–170.
46. Seitz M, Dewald B, Gerber N, Baggiolini M. Enhanced production of neutrophil-activating peptide-1/interleukin-8 in rheumatoid arthritis. *J Clin Invest* 1991; **87**: 463–469.
47. Krauss S. Response of serum haptoglobin to inflammation in adrenalectomized rat. *Proc Soc Exp Biol Med* 1962; **112**: 552–554.
48. Perretti M, Mugridge KG, Becherucci C, Parente L. Evidence that interleukin-1 and lipoxygenase metabolites mediate the lethal effect of complete Freund's adjuvant in adrenalectomized rats. *Lymphokine Cytokine Res* 1991; **10**: 239–243.
49. Butler LD, Layman NK, Riedl PE, *et al.* Neuroendocrine regulation of *in vivo* cytokine production and effects: I. *In vivo* regulatory networks involving the neuroendocrine system, interleukin-1 and TNF-$\alpha$. *J Neuroimmunol* 1989; **24**: 143–153.
50. Zuckerman SH, Shellaas J, Butler LD. Differential regulation of lipopolysaccharide-induced interleukin-1 and tumour necrosis factor synthesis: effects of endogenous and exogenous glucocorticoids and the role of the pituitary–adrenal axis. *Eur J Immunol* 1989; **19**: 301–305.
51. Parant M, Le Contel C, Parant F, Chedid L. Influence of endogenous glucocorticoid on endotoxin-induced production of circulating TNF-$\alpha$. *Lymphokine Cytokine Res* 1991; **10**: 265–271.
52. Perretti M, Becherucci C, Scapigliati G, Parente L. The effect of adrenalectomy on interleukin-1 release *in vitro* and *in vivo*. *Br J Pharmacol* 1989; **98**: 1137–1142.
53. De Simoni MG, Sironi M, De Luigi A, Manfridi A, Mantovani A, Ghezzi P. Intracerebroventricular injection of interleukin-1 induces high circulating levels of interleukin 6. *J Exp Med* 1990; **171**: 1773–1778.
54. Lazar GJ, Lazar G, Agarwal MK. Modification of septic shock in mice by the antiglucocorticoid RU 38486. *Circ Shock* 1992; **36**: 180–184.
55. Akahoshi T, Oppenheim JJ, Matsushima K. Induction of high-affinity interleukin-1 receptor on human peripheral blood lymphocytes by glucocorticoid hormones. *J Exp Med* 1988; **167**: 924–936.
56. Gottschall PE, Koves K, Mizuno K, Tatsuno I, Arimura A. Glucocorticoid upregulation on interleukin-1 receptor expression in a glioblastoma cell line. *Am J Physiol* 1991; **261**: E362–E368.
57. Bauer J, Lengyel G, Bauer TM, Acs G, Gerok W. Regulation of interleukin-6 receptor expression in human monocytes and hepatocytes. *FEBS Lett* 1989; **249**: 27–30.
58. Chambaut-Guerin AM, Thomopoulos P. Regulation by dexamethasone and 1,25-dihydroxyvitamin D3 of the 75 Kd-type A tumour necrosis factor receptor surface expression and mRNA level in human U937 cells. *Eur Cytokine Netw* 1991; **2**: 355–360.
59. Libert C, Van Bladel S, Brouckaert P, Fiers W. The influence of modulating substances on tumour necrosis factor and interleukin-6 levels after injection of murine tumour necrosis factor or lipopolysaccharide in mice. *J Immunother* 1991; **10**: 227–235.
60. Dinarello CA, Wolff SM. The role of interleukin-1 in disease. *N Engl J Med* 1993; **328**: 106–113.
61. Dayer J-M, de Rochemonteix B, Burrus B, Demczuk S, Dinarello CA. Human recombinant interleukin-1 stimulates collagenase and prostaglandin $E_2$ production by human synovial cells. *J Clin Invest* 1986; **77**: 645–648.
62. Flower RJ. Lipocortin and the mechanism of action of the glucocorticoids. *Br J Pharmacol* 1988; **94**: 987–1015.
63. Solito E, Parente L. Modulation of phospholipase $A_2$ activity in human fibroblasts. *Br J Pharmacol* 1989; **96**: 656–660.
64. Goppelt-Struebe M, Rehfeldt W. Glucocorticoids inhibit TNF-$\alpha$-induced cytosolic phospholipase $A_2$ activity. *Biochim Biophys Acta* 1992; **1127**: 163–167.
65. Kawada N, Mizoguchi Y, Shin T, *et al.* Interferon gamma stimulates prostaglandin $E_2$ production by mouse Kupffer cells. *Prostaglandins Leukot Essent Fatty Acids* 1990; **40**: 275–279.
66. Raz A, Wyche A, Siegel N, Needleman P. Regulation of fibroblast cyclooxygenase synthesis by interleukin-1. *J Biol Chem* 1988; **263**: 3022–3028.
67. Masferrer JL, Zweifel BS, Seibert K, Needleman P. Selective regulation of cellular cyclooxygenase by dexamethasone and endotoxin in mice. *J Clin Invest* 1990; **86**: 1375–1379.
68. Coyne DW, Nickols M, Bertrand W, Morrison AR. Regulation of mesangial cell cyclooxygenase synthesis by cytokines and glucocorticoids. *Am J Physiol* 1992; **263**: F97–F102.
69. Suffys P, Beyaert R, Van Roy F, Fiers W. Reduced tumour necrosis factor-induced cytotoxicity by inhibitors of the arachidonic acid metabolism. *Biochem Biophys Res Commun* 1987; **149**: 735–743.
70. Hepburn A, Boeynaems JM, Fiers W, Dumont JE. Modulation of TNF-$\alpha$ cytotoxicity in L929 cells by bacterial toxins, hydrocortisone and inhibitors of arachidonic acid metabolism. *Biochem Biophys Res Commun* 1987; **149**: 815–822.
71. Satomi N, Sakurai A, Haranaka R, Haranaka K. Preventive effects of several chemicals against lethality of recombinant human tumour necrosis factor. *J Biol Response Mod* 1988; **7**: 54–64.

72. Beyaert R, Suffys P, Van Roy F, Fiers W. Inhibition by glucocorticoids of tumour necrosis factor-mediated cytotoxicity. Evidence against lipocortin involvement. *FEBS Lett* 1990; **262**: 93–96.
73. Ribeiro RA, Flores CA, Cunha FQ, Ferreira SH. IL-8 causes *in vivo* neutrophil migration by a cell-dependent mechanism. *Immunology* 1991; **73**: 472–477.
74. Perretti M, Flower RJ. Modulation of IL-1-induced neutrophil migration by dexamethasone and lipocortin 1. *J Immunol* 1993; **150**: 992–999.
75. Maloff BL, Shaw JE, Di Meo TM. IL-1 dependent model of inflammation mediated by neutrophils. *J Pharmacol Meth* 1989; **22**: 133–140.
76. Colic M, Drabek D. Expression and function of intercellular adhesion molecule 1 (ICAM-1) on rat thymic macrophages in culture. *Immunol Lett* 1991; **28**: 251–257.
77. Detma M, Tenorio S, Hettmannsperger U, Ruszcak Z, Orfanos CE. Cytokine regulation of proliferation and ICAM-1 expression of human dermal microvascular endothelial cells *in vitro*. *J Invest Dermatol* 1992; **98**: 147–153.
78. Moncada S, Palmer RMJ, Higgs EA. Nitric oxide: physiology, pathophysiology, and pharmacology. *Pharmacol Rev* 1991; **43**: 109–141.
79. Pfeilschifter J, Schwarzenbach H. Interleukin-1 and tumour necrosis factor stimulate cGMP formation in rat renal mesangial cells. *FEBS Lett* 1990; **273**: 185–187.
80. Radomski MW, Palmer RM, Moncada S. Glucocorticoids inhibit the expression of an inducible, but not the constitutive, nitric oxide synthase in vascular endothelial cells. *Proc Natl Acad Sci USA* 1990; **87**: 10043–10047.
81. McCall TB, Palmer RM, Moncada S. Induction of nitric oxide synthase in rat peritoneal neutrophils and its inhibition by dexamethasone. *Eur J Immunol* 1991; **21**: 2523–2527.
82. Bertini R, Bianchi M, Ghezzi P. Adrenalectomy sensitizes mice to the lethal effects of interleukin-1 and tumour necrosis factor. *J Exp Med* 1988; **167**: 1708–1712.
83. Brouckaert P, Everaerdt B, Fiers W. The glucocorticoid antagonist RU38486 mimics interleukin-1 in its sensitization to the lethal and interleukin-6-inducing properties of tumour necrosis factor. *Eur J Immunol* 1992; **22**: 981–986.
84. Okusawa S, Gelfand JA, Ikejima T, Connolly RJ, Dinarello CA. Interleukin-1 induces a shock-like state in rabbits. Synergism with tumour necrosis factor and the effect of cyclooxygenase inhibition. *J Clin Invest* 1988; **81**: 1162–1172.
85. Coelho MM, Souza GE, Pela IR. Endotoxin-induced fever is modulated by endogenous glucocorticoids in rats. *Am J Physiol* 1992; **263**: R423–R427.
86. Toth LA, Gardiner TW, Krueger JM. Modulation of sleep by cortisone in normal and bacterially infected rabbits. *Am J Physiol* 1992; **263**: R1339–R1346.
87. Plata-Salaman CR. Dexamethasone inhibits food intake suppression induced by low doses of interleukin-1$\beta$ administered intracerebroventricularly. *Brain Res Bull* 1991; **27**: 737–738.
88. Pedersen P, Hasselgren PO, Li SJ, Hiyama DT, Fischer JE. Synthesis of acute-phase proteins in perfused liver following administration of recombinant interleukin-1$\alpha$ to normal or adrenalectomized rats. *J Surg Res* 1988; **45**: 333–341.
89. Rupp RG, Fuller GM. The effects of leucocytic and serum factors on fibrinogen biosynthesis in cultured hepatocytes. *Exp Cell Res* 1979; **118**: 23–30.
90. Okamoto H, Ohashi Y, Itoh N. Involvement of leukocyte and glucocorticoid in the acute-phase response of angiotensinogen. *Biochem Biophys Res Commun* 1987; **145**: 1225–1230.
91. Sobocinski PZ, Canterbury WJJ, Knutsen GL, Hauer EC. Effect of adrenalectomy on cadmium- and turpentine-induced hepatic synthesis of metallothionein and $\alpha_2$-macrofetoprotein in the rat. *Inflammation* 1981; **5**: 153–164.
92. Castell JV, Gomez-Lechon MJ, David M, Hirano T, Kishimoto T, Heinrich PC. Recombinant human interleukin-6 (IL-6/BSF-2/HSF) regulates the synthesis of acute phase proteins in human hepatocytes. *FEBS Lett* 1988; **232**: 347–350.
93. Baumann H, Wong GG. Hepatocyte-stimulating factor III shares structural and functional identity with leukemia-inhibitory factor. *J Immunol* 1989; **143**: 1163–1167.
94. Richards CD, Brown TJ, Shoyab M, Baumann H, Gauldie J. Recombinant oncostatin M stimulates the production of acute phase proteins in hepG2 cells and rat primary hepatocytes *in vitro*. *J Immunol* 1992; **148**: 1731–1736.
95. Borth W, Luger TA, Identification of $\alpha_2$-macroglobulin as a cytokine plasma protein. *J Biol Chem* 1989; **264**: 5818–5825.
96. Xia D, Samols D. Protective effects of rabbit C-reactive protein (RAB-CRP) against mediators of septic shock. *FASEB J* 1992; **6**: A1344.
97. Jaattela M, Ilvesmaki V, Voutilainen R, Stenman UH, Saksela E. Tumour necrosis factor as a potent inhibitor of adrenocorticotropin-induced cortisol production and steroidogenic P450 enzyme gene expression in cultured human fetal adrenal cells. *Endocrinology* 1991; **128**: 623–629.
98. Moore RN, Goodrum KJ, Berry LJ. Mediation of an endotoxic effect by macrophages. *J Reticuloendothel Soc* 1976; **19**: 187–197.
99. Moore RN, Goddrum KJ, Couch REJ, Berry LJ. Elicitation of endotoxemic effects in C3H/HeJ mice with glucocorticoid antagonizing factor and partial characterization of the factor. *Infect Immun* 1978; **19**: 79–86.
100. Stith RD, McCallum RE. Down regulation of hepatic glucocorticoid receptors after endotoxin treatment. *Infect Immun* 1983; **40**: 613–621.
101. Salkowski CA, Vogel SN. Lipopolysaccharide increases glucocorticoid receptor expression in murine macrophages. *J Immunol* 1992; **149**: 4041–4047.
102. Hill MR, Stith RD, McCallum RE. Interleukin-1: a regulatory role in glucocorticoid-regulated hepatic metabolism. *J Immunol* 1986; **137**: 858–862.
103. Besedovsky HO, Sorkin E, Keller M, Muller J. Changes in blood hormone levels during the immune response. *Proc Soc Exp Biol Med* 1975; **150**: 466–470.
104. Derijk R, Van Rooijen N, Tilders FJ, Besedovsky HO, Del Rey A, Berkenbosh F. Selective depletion of macrophages prevents pituitary-adrenal activation in response to subpyrogenic, but not pyrogenic, doses of bacterial endotoxin in rats. *Endocrinology* 1991; **129**: 330–338.
105. Besedowsky H, del Rey A, Sorkin E, Dinarello CA. Immunoregulatory feedback between interleukin-1 and glucocorticoid hormones. *Science* 1986; **233**: 652–654.
106. Rivier C, Chizzonite R, Vale W. In the mouse, the activation of the hypothalamic–pituitary–adrenal axis by a lipopolysaccharide (endotoxin) is mediated through interleukin-1. *Endocrinology* 1989; **125**: 2800–2805.
107. Mefford IN, Masters CF, Heyes MP, Eskay RL. Cytokine-induced activation of the neuroendocrine stress axis persists in endotoxin-tolerant mice. *Brain Res* 1991; **557**: 327–330.
108. Perlstein RS, Mougey EH, Jackson WE, Neta R. Interleukin-1 and interleukin-6 act synergistically to stimulate the release of adrenocorticotropic hormone *in vivo*. *Lymphokine Cytokine Res* 1991; **10**: 141–146.
109. Woloski BMRNJ, Smith EM, Meyer WJI, Fuller GM, Blalock JE. Corticotropin-releasing activity of monokines. *Science* 1985; **230**: 1035–1037.
110. Bernton EW, Beach JE, Holaday JW, Smallridge RC, Fein HG. Release of multiple hormones by a direct action of interleukin-1 on pituitary cells. *Science* 1987; **238**: 519–521.
111. Tsagarakis S, Gillies G, Rees LH, Besser M, Grossman A. Interleukin-1 directly stimulates the release of corticotropin releasing factor from rat hypothalamus. *Neuroendocrinology* 1989; **49**: 98–101.
112. Sapolsky R, Rivier C, Yamamoto G, Plotsky P, Vale W. Interleukin-1 stimulates the secretion of hypothalamic corticotropin-releasing factor. *Science* 1987; **238**: 522–524.
113. Berkenbosch F, van Oers J, del Rey A, Tilders F, Besedovsky H. Corticotropin-releasing factor-producing neurons in the rat activated by interleukin-1. *Science* 1987; **238**: 524–526.
114. Bernardini R, Calogero AE, Mauceri G, Chrousos GP. Rat hypothalamic corticotropin-releasing hormone secretion *in vitro* is stimulated by interleukin-1 in an eicosanoid-dependent manner. *Life Sci* 1990; **47**: 1601–1607.
115. Katsuura G, Gottschall PE, Dahl RR, Arimura A. Adrenocorticotropin release induced by intracerebroventricular injection of recombinant human interleukin-1 in rats: possible involvement of prostaglandin. *Endocrinology* 1988; **122**: 1737–1779.
116. Morimoto A, Murakami N, Nakamori T, Sakata Y, Watanabe T. Possible involvement of prostaglandin E in development of ACTH response in rats induced by human recombinant interleukin-1. *J Physiol* 1989; **411**: 245–256.
117. Weidenfeld J, Abramsky O, Ovadia H. Effect of interleukin-1 on ACTH and corticosterone secretion in dexamethasone and adrenalectomized pretreated male rats. *Neuroendocrinology* 1989; **50**: 650–654.
118. Brown R, Li Z, Vriend CY, *et al.* Suppression of splenic macrophage interleukin-1 secretion following intacerebroventricular injection of interleukin-1$\beta$: evidence for pituitary–adrenal and sympathetic control. *Cell Immunol* 1991; **132**: 84–93.
119. Sundar SK, Becker KJ, Cierpial MA, *et al.* Intracerebroventricular infusion of interleukin-1 rapidly decreases peripheral cellular immune responses. *Proc Natl Acad Sci USA* 1989; **86**: 6398–6402.
120. Sundar SK, Cierpial MA, Kilts C, Ritchie JC, Weiss JM. Brain IL-1-induced immunosuppression occurs through activation of both pituitary–adrenal axis and sympathetic nervous system by corticotropin-releasing factor. *J Neurosci* 1990; **10**: 3701–3706.
121. Ruddle NH, Bergman CM, McGrath KM, *et al.* An antibody to lymphotoxin and tumour necrosis factor prevents transfer of experimental allergic encephalomyelitis. *J Exp Med* 1990; **172**: 1193–1200.
122. Mason D. Genetic variation in the stress response: susceptibility to experimental allergic encephalomyelitis and implications for human inflammatory disease. *Immunol Today* 1991; **12**: 57–60.
123. Nakamura H, Motoyoshi S, Kadokawa T. Anti-inflammatory action of interleukin 1 through the pituitary–adrenal axis in rats. *Eur J Pharmacol* 1988; **151**: 67–73.
124. Sternberg EM, Hill JM, Chrousos GP, *et al.* Inflammatory mediator-induced hypothalamic–pituitary–adrenal axis activation is defective in streptococcal cell wall arthritis-susceptible Lewis rats. *Proc Natl Acad Sci USA* 1989; **86**: 2374–2378.
125. Sheagren JN. Septic shock and corticosteroids. *N Engl J Med* 1981; **305**: 456–457.
126. Veterans Administration Systemic Sepsis Cooperative Study Group. Effect of high-dose glucocorticoid therapy on mortality in patients with clinical signs of systemic sepsis. *N Engl J Med* 1987; **317**: 659–665.
127. Bone RC, Fisher CJJ, Clemmer TP, *et al.* A controlled clinical trial of high-dose methylprednisolone in the treatment of severe sepsis and septic shock. *N Engl J Med* 1987; **317**: 653–658.
128. Munoz C, Carlet J, Fitting C, Misset B, Blériot J-P, Cavaillon J-M.

Dysregulation of *in vitro* cytokine production by monocytes during sepsis. *J Clin Invest* 1991; **88**: 1747–1754.
129. Baker CC, Chaudry IH, Gaines HO, Baue AE. Evaluation of factors affecting mortality rate after sepsis in a murine cecal ligation and puncture model. *Surgery* 1983; **94**: 331–335.
130. Echtenacher B, Falk W, Mannel D, Krammer PH. Requirement of endogenous tumour necrosis factor/cachectin for recovery from experimental peritonitis. *J Immunol* 1990; **145**: 3762–3768.
131. van der Meer JWM, Barza M, Wolff SM, Dinarello CA. A low dose of recombinant interleukin-1 protects granulocytopenic mice from lethal Gram-negative infection. *Proc Natl Acad Sci USA* 1988; **85**: 1620–1623.
132. Ramilo O, Saez-Llorens X, Mertsola J, *et al.* Tumour necrosis factor α/cachectin and interleukin-1β initiate meningeal inflammation. *J Exp Med* 1990; **172**: 497–507.
133. Saukkonen K, Sande S, Cioffe C, Wolpe S, Sherry B, Cerami A. The role of cytokines in the generation of inflammation and tissue damage in experimental Gram-positive meningitis. *J Exp Med* 1990; **171**: 439–448.
134. Mustafa MM, Ramilo O, Saez-Llorens X, Olsen KD, Magness RR, McCracken GHJ. Cerebrospinal fluid prostaglandins, interleukin-1β, and tumour necrosis factor in bacterial meningitis. Clinical and laboratory correlations in placebo-treated and dexamethasone-treated patients. *Am J Dis Child* 1990; **144**: 883.

ACKNOWLEDGEMENT. G.F. is recipient of a fellowship from Consorzio Dompé/Biolaq, L'Aquila, Italy.

**Received 29 April 1993; accepted 3 May 1993**
